# Polyphenols in Pancreatic Cancer Management: Exploring the Roles and Mechanisms of Resveratrol and Epigallocatechin

**DOI:** 10.32604/or.2025.065222

**Published:** 2025-08-28

**Authors:** David A de la Garza-Kalife, Verónica L Loaiza-Gutiérrez, Esther Alhelí Hernández-Tobías, Carlos A González-Villarreal, Jose Francisco Islas, Michelle G Santoyo-Suárez, Elsa N Garza-Treviño, Paulina Delgado-Gonzalez

**Affiliations:** 1 Department of Biochemistry and Molecular Medicine, Faculty of Medicine, Autonomous University of Nuevo León (UANL), Monterrey, 64460, Mexico; 2 School of Public Health and Nutrition, Autonomous University of Nuevo León (UANL), Monterrey, 64460, Mexico; 3 Department of Basic Sciences, Laboratory of Molecular Genetics, University of Monterrey (UDEM), Monterrey, 66238, Mexico

**Keywords:** Pancreatic cancer (PC), polyphenols, resveratrol, epigallocatechin, bioactive compounds

## Abstract

Emerging evidence highlights the potential of bioactive compounds, particularly polyphenols, as adjunctive therapeutic agents in the treatment of pancreatic cancer (PC), one of the most aggressive malignancies. This review focuses on epigallocatechin gallate (EGCG) and resveratrol due to their extensively documented anticancer activity, favorable safety profiles, and their unique ability to modulate multiple signaling pathways relevant to pancreatic tumorigenesis. Among polyphenols, these two have shown superior anti-cancer activity, epigenetic regulatory effects, and synergy with standard chemotherapies in preclinical pancreatic cancer models. Resveratrol exhibits anti-proliferative effects by modulating key signaling pathways, including phosphatidylinositol 3 kinase (PI3K)/protein kinase B (Akt), nuclear factor kappa-B (NF-κB), and tumor protein 53 (p53). EGCG exerts anti-cancer activity by targeting multiple cellular processes, such as oxidative stress reduction, and suppression of inflammatory mediators like Interleukin-6 (IL-6) and Tumor Necrosis Factor-α (TNF-α). Both EGCG and resveratrol exert anti-pancreatic cancer effects partly through direct interactions with cell surface receptors and modulation of intracellular cascades. EGCG targets the 67 kDa laminin receptor (67LR), which is overexpressed in pancreatic cancer cells, triggering apoptosis, cyclic guanosine monophosphate (cGMP) production and activation of the PKCδ/acid sphingomyelinase (ASM) cascade. Resveratrol inhibits insulin-like growth factor-1 receptor (IGF-1R) activation of the PI3K/Akt and Wnt signaling pathways, while concurrently activating tumor suppressor p53. These interactions suppress proliferation, promote apoptosis, and reduce epithelial-mesenchymal transition (EMT), thereby limiting tumor progression. Both polyphenols enhance chemosensitivity and reduce resistance to conventional therapies, including gemcitabine, by modulating drug transporters and apoptotic pathways. Furthermore, their epigenetic influence, particularly via DNA methylation and histone modification, suggests a broader role in pancreatic cancer prevention. Understanding the roles and mechanisms of resveratrol and EGCG in pancreatic cancer provides valuable insights into novel treatment strategies. The integration of polyphenols into conventional therapeutic approaches may offer new hope for improving patient outcomes.

## 1 Introduction

Pancreatic cancer (PC) is one of the leading causes of cancer-related death worldwide, with 510,992 new cases and 467,409 deaths by 2022. PC is projected to surpass colorectal cancer as the second leading cause of cancer mortality by 2040, and only second behind lung cancer [1]. The most common and aggressive subtype is pancreatic ductal adenocarcinoma (PDAC), with 50%–55% of patients diagnosed with metastatic disease, resulting in a dismal 5-year survival rate of less than 10%. This prognosis has remained largely unchanged for decades [2–4].

Genetic factors, such as mutations in the *KRAS* oncogene, play a significant role in most PCs. Other risk factors include smoking, microbiome composition, and infection with *H. pylori* [5], red meat consumption, obesity, and diabetes [6]. Clinical symptoms typically appear at advanced stages of the disease and include jaundice, abdominal and back pain, nausea, venous thromboembolic events, weight loss, and anorexia, the latter being a marker of poor prognosis [7].

Treatment strategies vary based on tumor staging and typically include surgery, chemotherapy, radiotherapy, and palliative care. Standard chemotherapy regimens for resectable PC often involve a six-month course of modified leucovorin, 5-fluorouracil, irinotecan, and oxaliplatin (MFOLFIRINOX) [8], or gemcitabine and capecitabine after radical resection [9]. However, chemoresistance is a common challenge [10].

At diagnosis, PC patients often exhibit malnutrition and altered metabolism, particularly in glucose metabolism [11]. In advanced stages, many develop tumor cachexia, a condition marked by weight loss exceeding 5% over 6 months, accompanied by anorexia, muscle wasting, and systemic inflammation [12]. Cachexia is a catabolic state that increases nutrient demands and impairs treatment efficacy and quality of life [13]. The patient’s nutritional state and diet influence disease progression and treatment efficacy [11,14].

**Figure 1:**
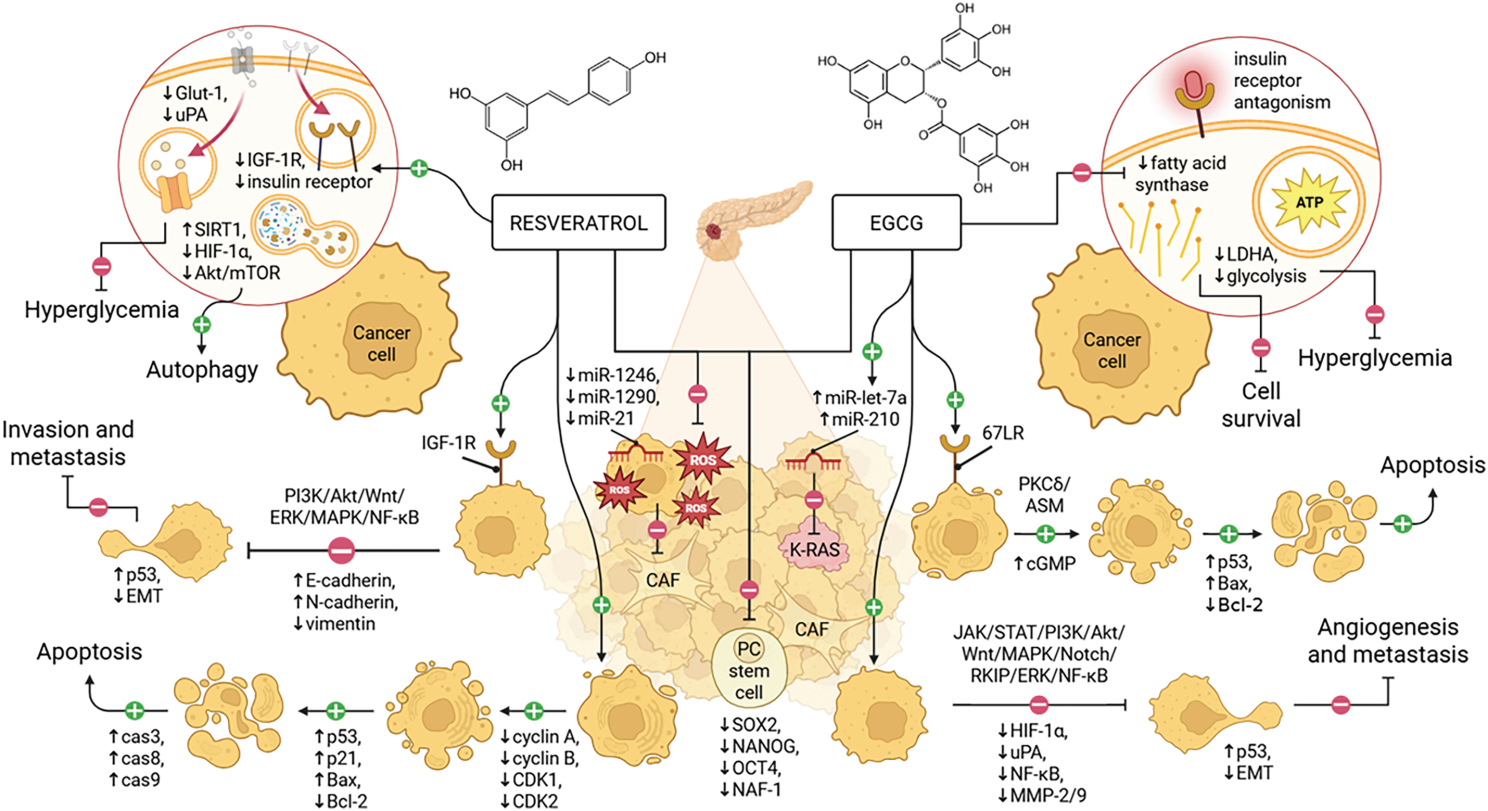
Resveratrol inhibits PC progression by promoting autophagy and apoptosis, targeting IGF-1R, altering EMT, and reducing metabolic stress and stemness. Similarly, EGCG acts through 67LR signaling to induce cell cycle arrest and apoptosis, modulating EMT, suppressing angiogenesis, and mitigating the same pathological features. EGCG, epigallocatechin gallate; PC, pancreatic cancer; CAF, cancer-associated fibroblast; EMT, epithelial–mesenchymal transition; IGF-1R, insulin-like growth factor 1 receptor; 67LR, 67-kDa laminin receptor. Adapted from Chouari et al. [15], and created in https://BioRender.com

Bioactive dietary compounds are well-known for their antioxidative effects and potential to modulate molecular pathways involved in cancer (Fig. 1). This review aims to explore the roles of resveratrol and epigallocatechin in the management of PC. Resveratrol is extensively studied and serves as a reference compound to explore EGCG, which shares several parallel mechanisms. Their well-documented, mechanistically diverse, and structurally distinct profiles provide a solid foundation for a focused, in-depth comparative analysis.

## 2 Cells and Mechanisms Involved in PC Progression

A hallmark of PC is oncogenic signaling driven by *KRAS* mutations, which, alongside other oncogenes and tumor suppressor genes like *SMAD4*, *CDKN2A*, *BRCA1*, *BRCA2*, *KDM6A*, *MYC*, and *TP53*, disrupt metabolic pathways. To test the effects of potentially therapeutic agents *in vitro*, researchers use human pancreatic carcinoma cell lines, e.g., AsPC-1 and BxPC-3, bearing the most common gene mutations in this type of cancer (*KRAS*, *TP53*, *CDKN2A/p16*, and *SMAD4/DPC4*) [16]. These alterations reprogram enzymes to support glycolysis and glutamine consumption, enabling the tumor to survive under high interstitial pressure, vascular collapse, and poor nutrient and oxygen diffusion resulting from excessive fibrosis (desmoplasia) [2,17,18]. All PDACs have somatic mutations in oncogenes and tumor suppressor genes affecting DNA repair, cellular proliferation pathways, and transcriptional activation of genes involved in cancer progression [19].

*KRAS* gene mutations are found in about 85% of cases, and they are critical for initiating and maintaining pancreatic tumors by encoding a Guanosine Triphosphatase (GTPase) enzyme that regulates essential intracellular signaling pathways, including rapidly accelerated fibrosarcoma (RAF)/mitogen-activated protein kinase (MAPK) and phosphatidylinositol 3 kinase (PI3K)/protein kinase B (Akt) [20,21]. The *KRAS* protein is activated through guanosine diphosphate-guanosine triphosphate (GDP-GTP) exchange via transmembrane receptors like epidermal growth factor receptor (EGFR) [21]. Another key gene in PDAC is *TP53*, which encodes the transcription factor p53, activated by DNA damage to regulate cell cycle checkpoints and promote DNA repair or apoptosis [22]. Mutations in *TP53*, found in up to 70% of PDACs, are linked to metastasis and activate the ADP-ribosylation factor 6/ArfGAP with SH3 domain, ankyrin repeat and PH domain 1p (ARF6/AMAP1) pathway, favoring malignancy in cooperation with *KRAS* mutations. This pathway influences tumor cell motility, programmed cell death ligand 1 (PD-L1) expression, and immune system evasion [23]. *SMAD4* gene inactivation is also common in PDAC and promotes tumor progression by inducing epithelial-to-mesenchymal transition (EMT), which confers migratory and invasive properties to cancer cells [24,25].

In addition to genetic alterations, PDAC features a dysregulated immune environment with tumorigenic regulatory T cells, intratumoral macrophages producing interleukin 6 (IL-6), and cancer-associated fibroblasts (CAFs) that promote tumor progression [26]. Pancreatic stellate cells (PSCs), critical for PDAC metabolism, contribute to the tumor microenvironment by secreting alanine for the tricarboxylic acid cycle, supporting non-essential amino acid and lipid biosynthesis [27]. Activated PSCs have a myofibroblast-like behavior, expressing α-smooth muscle actin (α-SMA), and can produce metabolic changes altering the tissue microenvironment by producing extracellular matrix (ECM) components such as type I and IV collagen, laminin, and fibronectin. The accumulation of these components causes extensive interstitial fibrosis and risk of PC by promoting tumor growth, immune escape, inflammatory response, and invasive metastasis promoted by EMT in cancer cells, indicated by their decreased expression of epithelial markers, such as E-cadherin, and increased expression of mesenchymal markers, such as vimentin and Snail [28]. Many cellular signals that contribute to PSC activation, as shown in Table 1, lead to tumor progression in PC, for example, hypoxia-inducible factor 1-alpha (HIF-1α) upregulates the hepatoma-derived growth factor gene under hypoxia, inhibiting PSC apoptosis and promoting the synthesis and deposition of ECM proteins [29]. The most potent activator reported was transforming growth factor-β (TGF-β), which can activate the MAPK signaling pathway, increasing the expression of mitogen-activated protein kinase 8 (JNK1) and extracellular signal-regulated kinase 1 (ERK1) [30].

**Table 1:** Cellular signals that contribute to PSC and PCSC activation

Cell	Activation mechanism	Signaling pathways	Cell function	References
	TGF-β	MAPK pathways enhance JNK1 and ERK1 expression	Differentiate PSCs into myofibroblasts, leading to ECM components, type I collagen and fibronectin production	[30]
	PDGF	PI3K and regulated protein kinase (ERK) pathways	Induces rapid activation of Raf-1, ERK1, and ERK2, as well as AP-1 protein, thereby promoting mitosis in PSCs	[40]
Pancreatic stellate cells (PSCs)	HIF-1a	Low oxygen availability exhibits an increased influx of calcium (Ca^2+^) through TRPC6 channels	Cellular stress evoked by hypoxia rapidly induces the transactivation of PSCs and induces their proliferation, perpetuating the fibrosis cycle	[29]
	IL-10	Anti-inflammatory pathways	Downregulates procollagen I and enhances collagenase gene expression	[30]
	miR-1246, miR-1290, miR-21	KRAS downstream effector pathways	Upregulates the expression of CAF-associated genes in PSCs and promotes migration of PDAC cells	[41]
	Hyperglycemia	ROS/TGFβ/SMAD	β-cell functional damage activates PSCs and promotes their proliferation, increasing ECM component production	[42]
	Hypercholesterolemia	JAK/STAT3	Induces the expression of various oncogenes via JAKs, IL-6, EGF, and Src family kinases	[33]
	Hedgehog	Promoted by overexpression of Ptch1 and Smo protein	Leads to PCSC proliferation, growth, survival, migration, metabolism, angiogenesis, and tumorigenesis	[32]
Pancreatic cancer stem cells (PCSCs)	mTOR	KRAS downstream effector pathways	Increases the availability of nutrients and metabolic regulation in the cellular growth of PCSCs	[39]
	Hyperglycemia	TGF-β	Increased PCSC development in a dose-dependent manner	[43]
	Hypercholesterolemia	Wnt/β-catenin	Maintains tumorigenesis and resistance to therapy	[44]

PC stem cells (PCSCs) display aberrant activation of multiple signaling pathways, including Hedgehog, Wnt, Notch, Janus kinase/signal transducer and activator of transcription (JAK/STAT), Nodal/Activin, and Hippo pathways, which lead to proliferation, increased tumor-inducing capacity and the ability to metastasize [31–33]. The SHH pathway [34], which participates in desmoplasia and metaplasia development with characteristics of pancreatic intraepithelial neoplasia [35], presents a 46-fold increase in the expression of SHH transcripts in CD44+CD24+ESA+ PCSCs when compared to PC cells or normal pancreatic epithelial cells without these markers [32]. Additionally, Huang et al. showed that cyclopamine-mediated hedgehog blockage reversed chemoresistance to gemcitabine in a PANC-1 PC cell line [36]. Other pathways reported to participate are TGF-β and Wnt/β-catenin, which are also involved in diabetes development, increasing the risk of PC [37]. The mammalian target of rapamycin (mTOR) pathway, which functions downstream of RAS, is crucial for the availability of nutrients and metabolic regulation in cellular growth. The activation of mTOR by PI3K class I varies in the liver, kidneys, and pancreas, which express the isoform PI3K-C2γ is expressed. The loss of PI3K-C2γ accelerates the development and progression of PDAC with hyperactivation of the mTOR pathway [38]. In addition, Matsubara et al. showed that the mTOR inhibitor, rapamycin, reduced the viability of PCSCs with different effects compared to cyclopamine, indicating distinct roles in which both pathways maintain PCSCs [39]. The relationship between dysregulated signaling pathways and PC activation is further illustrated in Table 1.

## 3 Metabolic Pathways in PC and Dietary Countereffects

PC cells exhibit disrupted metabolism of carbohydrates, lipids, and proteins, contributing to the onset and progression of the disease. Alterations in lipid metabolism support membrane synthesis during cell proliferation and energy storage during metabolic stress, promoting resistance to chemotherapy [45]. CD36, a receptor involved in fatty acid uptake, is linked to tumor size and prognosis, with decreased expression correlating with larger tumors and shorter survival [46]. Conversely, increased CD36 expression is associated with resistance to gemcitabine. Fatty acid oxidation may also contribute to chemoresistance in PCSCs [47], exacerbating cachexia and oxidative stress, and fostering a genotoxic environment that aids cell migration, proliferation, and apoptosis [48]. Additionally, altered cholesterol metabolism promotes carcinogenesis, with low-density lipoprotein (LDL) activation of the signal transducer and activator of transcription 3 (STAT3) pathway enhancing PC cell survival, migration, and invasion [33].

Most patients with PDAC present with glucose metabolism alterations, including diabetes, before diagnosis [49]. This abnormal metabolism affects nutritional status and prognosis. Elevated glucose levels, which promote the formation of advanced glycation end products (AGEs), lead to inflammation and oxidative stress, contributing to cancer progression [50,51]. PC cells rely on aerobic glycolysis to produce lactic acid, which boosts resistance to chemotherapy, radiotherapy, and immune responses [52]. Hyperglycemia, insulin resistance, and chronic inflammation further exacerbate the aggressiveness of the tumor [53].

Amino acid metabolism also plays a crucial role in PDAC progression. Glutamine is essential for cancer cell survival, and its deprivation has been linked to the induction of EMT [54]. Increased branched-chain amino acids (BCAA) in systemic and intracellular levels are also associated with tumor progression [55].

Nutritional interventions are critical in managing PDAC, aiming to address the hypercatabolic state through a high intake of macronutrients [14]. Due to cancer’s inflammatory nature and oxidative stress, dietary strategies involving antioxidants, such as the Mediterranean diet, rich in bioactive compounds like Resveratrol and Epigallocatechin, have been proposed to complement cancer treatment [56]. These compounds have been shown to modulate multiple signaling pathways relevant to PC, namely insulin-like growth factor, Wnt, Notch, JAK-STAT, and mTOR, providing therapeutic potential for PDAC [57,58].

## 4 Polyphenols and PC

Polyphenols are polyaromatic compounds and potent natural antioxidants that prevent free radical formation [59]. These compounds target cancer, including metastasis, angiogenesis, and chemoresistance [60]. Due to their antioxidant activity, known low adverse effects, and potential for combining with traditional chemotherapy, polyphenols are cost-effective therapeutic agents for PC [60].

A review of 74 studies on phytochemicals concluded that factors like phytochemical type, concentration, and cell line determine cell viability and apoptosis more effectively than other variables [61]. Natural compounds like paclitaxel and vincristine have already been integrated into clinical practice [62]. However, the low survival rates in PC remain a challenge, partly due to drug resistance, which polyphenols may help counteract [62,63].

### 4.1 Benefits of Resveratrol in Cancer

Resveratrol, a polyphenol in the stilbene family, is found in grapes, wine, peanuts, and berries [60,64,65], has attracted attention due to its beneficial effects on cardiovascular health [66]. Beyond cardiovascular protection, resveratrol exhibits anti-inflammatory, antioxidant, and anticarcinogenic properties, making it an attractive candidate for cancer therapy [64,65]. Studies show that resveratrol inhibits the growth of various cancer cells, including PC, by modulating pathways related to apoptosis, autophagy, oxidative stress, and chemoresistance [60].

The anticancer effects of resveratrol are mediated through multiple molecular pathways, including epigenetic modifications, induction of autophagic cell death via activated sirtuin 1 (SIRT1) and Akt/mTOR pathway inhibition, and modulation of autophagy-related effectors like Akt and SIRT1, while also influencing EMT markers such as E-cadherin, N-cadherin, and vimentin in the presence of senescent CAFs [67].

*In vitro* studies have shown that resveratrol inhibits human PC cell proliferation in a dose-dependent manner [65]. Its effects include antioxidation, anti-inflammatory actions, and inhibition of cyclooxygenase, which disrupts tumor initiation and progression [68]. In animal models, resveratrol modulates NF-κB, which is associated with tumor proliferation, invasion, and metastasis, thus slowing the progression of precancerous lesions [69]. Resveratrol also reduces inflammatory responses in pancreatitis and precancerous lesion formation by directly modulating cytokine generation [70]. Furthermore, resveratrol has been shown to mitigate oxidative stress and reduce nicotine-induced proliferation in PC cells [71]. In mouse models, resveratrol (50 μM) inhibited the hypoxic tumor microenvironment induced by PSC fibrosis via HIF-1α, reducing tumor progression [72].

### 4.2 Benefits of Epigallocatechin in Cancer

Catechins are a group of polyphenols including epigallocatechin gallate (EGCG), epigallocatechin (EGC), epicatechin gallate (ECG), gallocatechin (GC), and catechin [73]. They are composed of a polyphenolic structure differentiated by the number of hydroxyl groups that allow them to exert their antioxidant, anti-tumor, and anti-inflammatory functions, explaining how they counteract various diseases, including different types of cancer [74,75]. EGCG is the most prevalent catechin in green tea, and it is also found in coffee, berries, grapes, and black tea [68,76]. Most studies have found that the main bioactive compound with effects against cancer is EGCG and that its impact depends on the dose and exposure time; however, the ways to exert these effects are quite varied.

EGCG is the most biologically active polyphenol found in green tea, and it has antioxidant, antiproliferative, and anti-tumor effects that help prevent tumor recurrence [75,77,78]. Previous studies have associated the daily number of cups of green tea consumed with the risk of developing cancer, showing a decrease in gastric cancer and PC risk with >5 cups and >4 cups per day, respectively [79]. Its structural characteristics contribute to its increased antioxidant, anti-inflammatory, and anticancer activities [80]. The anticancer effects of EGCG are mediated through multiple molecular pathways, including: (1) activation of apoptotic pathways by enhancing caspase-3 activity, cytochrome c release, and PARP cleavage; (2) epigenetic regulation via DNMT inhibition and histone modification, reactivating tumor suppressor genes like p16, PTEN, and p53; (3) inhibition of angiogenesis and metastasis by blocking HIF-1α, NF-κB, MMP-2/9, and uPA; (4) modulation of the tumor microenvironment through interaction with the overexpressed 67 kDa laminin receptor (67LR); (5) regulation of key signaling pathways such as JAK/STAT, PI3K/Akt, MAPK, Wnt, and Notch to inhibit proliferation and induce cancer cell death; (6) modulation of oncogenic microRNAs including miR-210, miR-203, and miR-125b; (7) inhibition of glycolytic enzymes, reducing cancer cell metabolism; and (8) enhancement of chemotherapy efficacy while reducing associated side effects [81,82].

As with previously mentioned resveratrol, EGCG has an inhibitory effect against NF-κB, a transcription factor that regulates genes acting on immunity and inflammation [83]. Fatty acid synthase, the key enzyme for converting dietary carbohydrates into fatty acids, is targeted by EGCG, which reduces tumor growth, induces cell cycle arrest, suppresses oncogene transcription, and enhances chemotherapy-induced cancer cell death by limiting saturated fatty acids synthesis [84]. A decrease in viability and proliferation has also been found in cultures with PC cell lines MIAPaCa-2 and SU.86.86 treated with EGCG at concentrations between 10–100 μM, finding an improved effect at high concentrations [83]. It has been reported that EGCG can inhibit the self-renewal of PCSCs by decreasing the activity of TCF/LEF, which are factors that favor the transcription of target genes for *WNT* [85] and transcription factor GLI, which are considered effectors of the Hedgehog signaling pathway [86]. Hu et al. found that EGCG functions as an insulin receptor antagonist, which can prevent PC cell stimulation by intra-pancreatic insulin [87]. A recent study indicated that EGCG changed the gene expression of the intrinsic apoptotic pathway (BAX, BAK, APAF1, Bcl-2, and Bcl-xL) in a p53-dependent and -independent manner, leading to cell apoptosis and inhibition of cell growth in PA-TU-8902, CFPAC-1, and CAPAN-1 human PC cell lines [88]. Studies in humans have only been performed with green tea, but they provide a first step toward studying this compound as an adjuvant in PC treatment.

### 4.3 Resveratrol and PC

Resveratrol exerts potent anticancer effects in part by targeting the insulin-like growth factor 1 receptor (IGF-1R), a key mediator of pancreatic cancer growth and metastasis [89]. Even in the presence of IGF-1, resveratrol suppresses cancer cell proliferation and promotes apoptosis by inhibiting IGF-1R-mediated activation of the PI3K/Akt and Wnt signaling pathways, while concurrently activating tumor suppressor p53 [89]. In pancreatic cancer cell lines such as PANC-1 and HPAC, downregulation of IGF-1R not only impairs proliferative and metastatic signaling through the MAPK and JAK/STAT pathways but also reduces EMT and insulin receptor β expression [90]. These findings support the therapeutic potential of resveratrol in targeting IGF-1R-driven oncogenic signaling in pancreatic cancer.

Yan et al. performed *in vitro* studies with human PSCs isolated from humans that showed resveratrol inhibits proliferation by more than 50% when exposed to doses of 200 μM for 72 h, and it inhibits invasion and migration by suppressing activity mediated by reactive oxygen species (ROS)/miR-21 and glycolysis in PC cells [91]. Other *in vitro* assays using Panc-1 and BxPC-3 cell lines exposed to 50 μM and 100 μM of resveratrol for 24 h, in a subcutaneous xenograft model of PC treated with doses of 50 mg/kg of resveratrol, have shown a decrease in stem cell markers (sex determining region Y)-box 2 (SOX2), nanog homeobox (NANOG), organic cation/carnitine transporter 4 (OCT4), resulting in the reduction of expression of nutrient-deprivation autophagy factor 1 (NAF-1), a genetic locus expressed in PC tissue which correlates with progression [92]. Srivani et al. characterized the interaction between resveratrol and HIF-1α, a regulator induced by lack of oxygen that is involved in several signaling pathways central for tumor development, migration, and metastasis, finding that its expression in PC cells decreased when treated with resveratrol [93]. Another study found that resveratrol inhibits the growth of tumor cells under chronic stress via the HIF-1α axis and the β2-adrenergic receptor-2, which can contribute to tumorigenesis and cancer development [94]. Cao et al. found that resveratrol inhibits the hyperglycemia-induced activity of PC cells, including the production of ROS and H_2_O_2_, expression of urokinase-type plasminogen activator (uPA), E-cadherin, and glucose transporter 1 (Glut-1), and activation of ERK, p38 MAPK, and NF-κB signaling pathways, indicating mechanisms for the chemoprevention of PC [95]. Western blot analysis of PC cells BxPC-3 treated with resveratrol nanoparticles implies that the compound executes cell apoptosis by downregulating cyclin A, cyclin B, cyclin-dependent kinase 1 (CDK1) and cyclin-dependent kinase 2 (CDK2), and upregulating p53 and p21 expressions, accompanied by enhancing cytochrome C expression, decreasing Bcl-2 expression, increasing Bax expression, and leading to the elevation of caspase-8, caspase-9, and caspase-3 activities [96]. Ratajczak et al. analyzed the antiproliferative and pro-apoptotic mechanisms of resveratrol on three human PC cell lines (EPP85-181P, EPP85-181RNO, and AsPC-1), as well as the normal pancreatic cell line H6c7, concluding that resveratrol affects the levels of the anti-apoptotic protein Bcl-2, significantly decreasing in a dose-dependent manner in the tested cancer lines and remaining unchanged in the normal pancreatic cell line [97].

Despite showing significant effects against cancer, resveratrol is limited by its low bioavailability. Studying other stilbene compounds like pterostilbene [98] and triacetyl resveratrol [99], which show promising results regarding lipophilicity, oral absorption, cellular uptake, and their half-life when compared with resveratrol, may offer a resveratrol analog with potentially better pharmacokinetic characteristics [100]. Pterostilbene was further found to decrease blood levels of adrenocorticotropic hormone (ACTH) and corticosterone, thus downregulating the glucocorticoid receptor, nuclear factor erythroid 2-related factor 2 (Nrf2)-dependent signaling, and antioxidant defenses in human PC-bearing mice [16]. A recent study found that triacetyl resveratrol suppressed PC growth by targeting the SHH pathway and modulating cyclin D1 and Bcl-2 expression, inhibiting EMT through the upregulation of miR-200 family members (miR-200a, miR-200b, and miR-200c) [101]. MiR-200 plays crucial roles in cancer initiation and metastasis, and its loss, typically a late event in pancreatic cancer progression, may contribute to the development of distant metastases [102]. Investigating resveratrol analogs has also provided better selectivity index values toward PC cell lines, as one study found that newly synthesized resveratrol analogs consistently reduced the PC cell subpopulation with a CD133^+^EpCAM^+^ stem-like phenotype while maintaining negligible toxicity against normal HFF-1 cells [100]. One study found that piceatannol, but not its analog resveratrol, reduced cancer-associated lipolysis by at least 50% in both cancer-conditioned media and cytokine-induced lipolysis *in vitro*, protecting tumor-bearing mice against cancer-associated cachexia [103].

Moreover, while phytochemicals alone have been proven to prevent cancer, natural diets include various compounds, and their combinations could modulate signaling pathways more efficiently, as Cykowiak et al. found studying the effect of isothiocyanate, xanthohumol, indole-3-carbinol, resveratrol, and their combinations on the Nrf2 signaling pathway of human PC cells [104]. Another study testing the cytotoxic effect of resveratrol, capsaicin, piceatannol, and sulforaphane in human tumor pancreatic cell lines also found that although each bioactive component used alone did not affect tumor growth, treatment with a combination of resveratrol analogs and capsaicin diminished tumor mass *in vivo* model [105].

Searching for potential therapeutic uses, Borska et al. found that resveratrol inhibited the proliferation of human PC cell lines with resistance to daunorubicin and mitoxantrone through phase-specific cell cycle arrest, depending on the type of cancer cells, supporting the possibility of using resveratrol to break chemotherapy resistance [106]. Furthermore, Barros et al. found that the viability of PANC-1 cells was more affected when doxorubicin and resveratrol combinations contained higher contents of resveratrol, explained by the ability of resveratrol to reduce the P-glycoprotein-mediated efflux of doxorubicin [107]. The combination of resveratrol and gemcitabine also showed significantly increased cell death and apoptosis of PC cells *in vitro* and inhibition of tumor growth *in vivo* compared to resveratrol or gemcitabine alone treatment [108]. The mechanism by which resveratrol enhances chemosensitivity to gemcitabine in PC cells may be through sterol regulatory element binding protein 1 (SREBP1) inhibition, evidenced by proliferating cell nuclear antigen inhibition, Bax expression, and increased apoptosis induced by gemcitabine [109].

Other studies found that resveratrol enhances gemcitabine sensitivity by downregulating NAF-1, inducing cellular ROS accumulation, and activating Nrf2 signaling pathways, shown by increased apoptosis and decreased PC cell proliferation in NAF-1 knockdown models [110]. Furthermore, resveratrol enhanced gemcitabine sensitivity by activating AMP-activated protein kinase (AMPK) and, thus, inducing YES-activated protein (YAP) cytoplasmic retention, Ser127 phosphorylation, and YAP transcriptional activity inhibition [111]. Pterostilbene also promoted chemosensitivity by inducing S-phase cell cycle arrest, apoptosis, and autophagic cell death and by inhibiting multidrug resistance protein 1 (MDR1) expression, downregulating receptor for advanced glycation end-products (RAGE)/PI3K/Akt signaling in gemcitabine-resistant PC cells [112]. Chen et al. identified a synthetic analog of resveratrol termed trans-4,4^′^-dihydroxystilbene (DHS) that inhibits DNA replication by targeting ribonucleotide reductase, the same enzyme that gemcitabine inhibits, and they found that DHS overcomes gemcitabine resistance in mouse models, demonstrating therapeutic potential for resistance to this drug [113]. Lastly, research into resveratrol-loaded nanoparticles shows great promise in improving the bioavailability, stability, and intracellular delivery of resveratrol while minimizing side effects, as presented by the results of Firouzi Amandi et al. [114]. Further research into resveratrol chemosensitization, analogs with better chemical profiles, and targeted delivery through nanotechnology may improve chemoprevention and treatment for patients with PC.

### 4.4 Epigallocatechin and PC

EGCG exerts anticancer effects through interaction with the 67-kDa laminin receptor (67LR), a cell surface protein overexpressed in several malignancies, including pancreatic cancer [115,116]. Acting as a natural ligand, EGCG binds 67LR to initiate a cancer-specific cell death pathway involving cyclic guanosine monophosphate (cGMP) production and subsequent activation of the PKCδ/acid sphingomyelinase (ASM) cascade. This signaling axis positions 67LR as a functional death receptor in tumor cells, and the EGCG-67LR interaction as a potential therapeutic target [117]. The specificity of this mechanism underscores the promise of EGCG or EGCG-mimetic compounds in selectively inducing apoptosis in cancer cells via 67LR-dependent pathways.

EGCG has been reported to inhibit cell growth and induce apoptosis in PC cells, such as MIA PaCa-2 and PANC-1 cells, by modulating cyclin D1 and cyclin-dependent kinases [118]. This induction of apoptosis is mediated by the activation of caspases 3, 8, and 9, and inhibition of the anti-apoptotic proteins Bcl-2 and Bcl-XL, as well as overexpression of pro-apoptotic agents like Bax and PUMA [119]. Studies further confirmed that EGCG modulates cell cycle proteins in other PC cell lines (PancTu-I, PANC-1, and PANC-89) [120]. MicroRNA let-7a, a post-transcriptional regulator of K-RAS involved in terminal differentiation and tumor suppression, is downregulated in cancers with poor prognosis, and studies show that combined treatment with sulforaphane, quercetin, and EGCG extract induces miR-let-7a expression and inhibits K-RAS in highly tumorigenic MIA-PaCa2 cell lines [121].

Shankar et al. found that EGCG inhibits the growth of human pancreatic tumors implanted in mice by reducing the phosphorylation of ERK, PI3K, Akt, and forkhead transcription factor like 1/forkhead box O3a (FKHRL1/FOXO3a), and modulating FOXO target genes [122]. EGCG also inhibits inflammatory pathways, including NF-κB in PC cells, decreasing the secretion of pro-inflammatory proteins like IL-6 and IL-8 [120]. In addition, EGCG inhibits lactate dehydrogenase A (LDHA), a key enzyme involved in cancer cell glycolysis, leading to reduced lactate production, glucose consumption, and glycolytic activity [123]. EGCG inhibits invasive metastasis in pancreatic adenocarcinoma by regulating the RAF kinase inhibitor protein (RKIP)/ERK/NF-κB pathway [124]. When combined with gemcitabine, EGCG suppressed tumor migration and invasion by downregulating the zinc finger E-box binding homeobox 1 (ZEB1), β-Catenin, and vimentin pathways, while inhibiting Akt and EMT pathways [125].

Clinical trials on EGCG for PC treatment are lacking, but epidemiological studies suggest that regular green tea consumption may reduce the risk of PC, with some studies reporting higher significance in women [126,127]. Nonetheless, further randomized clinical trials are necessary to establish the role of EGCG as an adjunct or stand-alone treatment in PC management.

On the other hand, although studies suggest an antiapoptotic effect of EGCG in ASPC-1 and BxPC-3 cells, this bioactive compound only inhibited the phosphorylation of focal adhesion kinase (FAK) and IGF-1R with no effect on apoptosis even at the highest levels [128]. Anti-proliferative effects have been observed in MIA PaCa-2, Panc-1, BxPC-3, and AsPC-1 cells mainly due to the binding of EGCG to the heat shock protein 90 (HsP90) proteins, which prevents their association with their chaperones and induces their degradation, coupled with the activation of caspase 3 [128,129]. Similarly, in Colo357 cells, EGCG induced the underexpression of interleukin-1 receptor, type I (IL-1RI), probably by inhibiting NF-κB with a marked decrease in cell viability mediated by pro-inflammatory IL-6 and proangiogenic interleukin 8 (IL-8) [130,131].

Studies in animal models are still scarce. The first reports are of xenograft models with subcutaneous injections of AsPC-1 cells, where a reduction in volume, proliferation, angiogenesis, metastasis, induction of apoptosis, as well as cell arrest, was observed when treated with EGCG [132]. On the contrary, others have reported that suffering from this condition was associated with consuming more than 5 cups of green tea a day [133]. The machine learning study conducted by Genc et al. supported the effects of resveratrol on cancer but found that catechin was not cytotoxic to PC cells at all concentrations, irrespective of the treatment time [61]. Correspondingly, the most recent meta-analysis of all the epidemiological evidence revealed that green tea is unrelated to PC [134]. Nevertheless, epidemiological studies do not allow for testing the effects of substances with appropriate adjustments in doses and study subjects, therefore, it is imperative to develop clinical trials on EGCG as an adjuvant treatment of PC.

Inhibition of cell migration and invasion has been observed mainly in combined therapies. In this sense, treatment in MIA PaCa-2 cells of bleomycin with EGCG for 72 h induced mitochondrial depolarization and cell arrest in the S phase of the cell cycle [135]. In parallel, it has been reported that peritoneal administration to this animal model enhanced the effect of gemcitabine, which reduced tumor growth by 67% when compared to controls, 27% and 15% more than EGCG and gemcitabine alone, respectively [136]. Likewise, a recent study indicated that the use of gemcitabine with EGCG in MIA-PaCa-2 and PANC-1 cells enhanced the effect of the drug by suppressing the phosphorylation of IGFR and inducing the degradation of Akt [125]. Another study on chemosensitization found that EGCG reduced ERK phosphorylation concentration-dependently, and sensitized gemcitabine, fluorouracil (5-FU), and doxorubicin to further suppress ERK phosphorylation in multiple cancer cell lines, including PC cells [137]. Cunha et al. showed that EGCG conjugated in a nanosystem-based nanoparticle enhanced BxPC3 apoptosis compared with EGCG alone, presenting the possibility of using nano vehicles to enhance the efficacy of EGCG [138]. Using EGCG to improve chemotherapy and diminish the doses and toxicity of current treatments calls for further research into safe, naturally available compounds.

## 5 Conclusions

Polyphenols, such as resveratrol and epigallocatechin, show considerable potential for preventing and treating PC. Their anti-inflammatory, antioxidant, and anti-tumor effects intervene at various stages of tumor progression, potentially reducing cancer cell proliferation, migration, and invasion while promoting apoptosis. PC cells overexpress specific targets like 67LR and IGF-1R, making them susceptible to polyphenols such as EGCG and resveratrol. These receptors are less active in normal cells, which may contribute to the cancer-specific effects and lower toxicity of these compounds. Both resveratrol and EGCG target molecular pathways involved in PC proliferation, metastasis, and treatment resistance, offering potential as chemosensitizers.

Although *in vitro* and *in vivo* studies demonstrate their efficacy, clinical applications are hindered by challenges such as low bioavailability, stability, and variability across cancer subtypes. Research into analogs and advanced delivery systems, such as nanotechnology, may help overcome these limitations. However, more clinical trials are needed to evaluate the effectiveness of polyphenols in treating PC and comparing them to current therapies.

Given the complexity of PC, combining polyphenols with existing therapies could improve efficacy and reduce recurrence. In conclusion, bioactive compounds like polyphenols hold significant potential as adjunct therapies and further research is needed to determine their role in transforming PC treatment paradigms and improving patient outcomes.

## Data Availability

Not applicable.

## Abbreviations

5-FU: Fluorouracil
67LR: 67-kDa laminin receptor
ACTH: Adrenocorticotropic hormone
AGEs: Advanced glycation end products
Akt: Protein kinase B
AMAP1: ArfGAP with SH3 domain, ankyrin repeat and PH domain 1p
AMPK: AMP-activated protein kinase
ARF6: ADP-ribosylation factor 6
ASM: Acid sphingomyelinase
BCAA: Branched chain amino acids
CAFs: Cancer-associated fibroblast
CDK1: Cyclin-dependent kinase 1
CDK2: Cyclin-dependent kinase 2
cGMP: Cyclic guanosine monophosphate
DNA: Deoxyribonucleic acid
ECG: Epicatechin gallate
ECM: Extracellular matrix
EGC: Epigallocatechin
EGCG: Epigallocatechin gallate
EGFR: Epidermal growth factor receptor
EMT: Epithelial–mesenchymal transition
ERK1: Extracellular signal-regulated kinase 1
FAK: Focal adhesion kinase
FKHRL1: Forkhead transcription factor like 1
FOXO3a: Forkhead box O3a
GC: Gallocatechin
GDP-GTP: guanosine diphosphate-guanosine triphosphate
Glut-1: Glucose transporter 1
GTPase: Guanosine Triphosphatase
HIF-1α: Hypoxia-inducible factor 1-alpha
HsP90: Heat shock protein 90
IGF-1R: Insulin-like growth factor 1 receptor
IL-1RI: Interleukin 1 receptor, type I
IL-6: Interleukin 6
IL-8: Interleukin 8
JAK: Janus kinase
JNK1: Mitogen-activated protein kinase 8
LDL: Low-density lipoprotein
MAPK: Mitogen-activated protein kinase
MDR1: Multidrug resistance protein 1
NANOG: Nanog homeobox
Nrf2: Nuclear factor erythroid 2-related factor 2
OCT4: Organic cation/carnitine transporter 4
PC: Pancreatic cancer
PCSCs: Pancreatic cancer stem cells
PDAC: Pancreatic ductal adenocarcinoma
PD-L1: Programmed cell death ligand 1
PI3K: Phosphatidylinositol 3 kinase
PSCs: Pancreatic stellate cells
RAF: Rapidly accelerated fibrosarcoma
RAGE: Receptor for advanced glycation end-products
RKIP: RAF kinase inhibitor protein
ROS: Reactive oxygen species
SIRT1: Activated sirtuin 1
SOX2: (Sex determining region Y)-box 2
SREBP1: Sterol regulatory element binding protein 1
STAT: Signal transducer and activator of transcription
STAT3: Signal transducer and activator of transcription 3
uPA: Urokinase-type plasminogen activator
ZEB1: Zinc finger E-box binding homeobox 1
